# Authors’ Reply: The University Medicine Greifswald’s Trusted Third Party Dispatcher: State-of-the-Art Perspective Into Comprehensive Architectures and Complex Research Workflows

**DOI:** 10.2196/67429

**Published:** 2024-11-29

**Authors:** Eric Wündisch, Peter Hufnagl, Peter Brunecker, Sophie Meier zu Ummeln, Sarah Träger, Fabian Prasser, Joachim Weber

**Affiliations:** 1 Core Unit Treuhandstelle, Berlin Institute of Health at Charité – Universitätsmedizin Berlin, Charitéplatz 1, Berlin, 10117, Germany, 49 1523 1394295; 2 Digital Pathology, Charité – Universitätsmedizin Berlin, Berlin, Germany; 3 Core Unit Research IT, Berlin Institute of Health at Charité – Universitätsmedizin Berlin, Berlin, Germany; 4 Medical Informatics Group, Center of Health Data Science, Berlin Institute of Health at Charité – Universitätsmedizin Berlin, Berlin, Germany; 5 Center for Stroke Research Berlin, Charité – Universitätsmedizin Berlin, Berlin, Germany; 6 German Centre for Cardiovascular Research (DZHK), Berlin, Germany

**Keywords:** architecture, scalability, trusted third party, application, security, consent, identifying data, infrastructure, modular, software, implementation, user interface, health platform, data management, data privacy, health record, electronic health record, EHR, pseudonymization

We would like to thank the authors of the letter [1] for their comments and for taking the time to engage with our work [2]. Their remarks provide us with the opportunity to clarify a key aspect of our article, and we are pleased to address the points they raised.

Most of the comments from the authors refer to functionalities provided by the TTP Dispatcher component. As mentioned in our article, our work focused on building a trusted third party (TTP) platform using *open* components, that is, software that is publicly available to the community [2]. For this reason, we built our platform around the core components Enterprise Identifier Cross-Referencing (E-PIX), Generic Pseudonym Administration Service (gPAS), and Generic Informed Consent Service (gICS), and we are grateful that these components are available as open versions. Consequently, we only covered the functionalities supported by these core components in the *Requirements* section. However, we did describe the TTP Dispatcher in the *Comparison With Prior Work* section and explicitly noted that it supports some of the functionalities we implemented, though not as open software at the time of writing.

Regarding authentication and authorization, we would like to note that the second table in our article [2] describes additional requirements that we elicited, which apply not only to the core components but also to the broader platform we envisioned [2]. Consequently, these requirements are not limited to the core components, and we note that E-PIX, gPAS, and gICS are not mentioned in the table. For example, the *User interfaces and services* requirements category, which lists the need for a “[c]ommon authentication and authorization framework with single-sign-on and associated rights and roles with the ability to connect to institutional directory services,” also includes the requirement for an “[i]ntegrated user interface across all services.” The Keycloak-based authentication and authorization mechanism we describe in the paper integrates with this user interface as well [2].

The additional information provided in the letter regarding the TTP Dispatcher’s functionalities and Keycloak support for E-PIX, gPAS, and gICS could be valuable to interested readers of our article, and we thank the authors for offering these insights. We look forward to further exchanges with the authors on the topic of TTP services, including the adoption of HL7 FHIR (Health Level Seven International Fast Healthcare Interoperability Resources).

## Abbreviations

E-PIX: Enterprise Identifier Cross-Referencing
gICS: Generic Informed Consent Service
gPAS: Generic Pseudonym Administration Service
HL7 FHIR: Health Level Seven International Fast Healthcare Interoperability Resources
TTP: trusted third party
